# The pharmacological and clinical aspects behind dose loading of biological disease modifying anti-rheumatic drugs (bDMARDs) in auto-immune rheumatic diseases (AIRDs): rationale and systematic narrative review of clinical evidence

**DOI:** 10.1186/s41927-020-00130-x

**Published:** 2020-07-28

**Authors:** Gerlienke E. Geurts-Voerman, Lise M. Verhoef, Bart J. F. van den Bemt, Alfons A. den Broeder

**Affiliations:** 1 Department of Rheumatology, Sint Maartenskliniek, PO Box 9011, 6500 GM Nijmegen, The Netherlands; 2 Department of Pharmacy, Sint Maartenskliniek, PO Box 9011, 6500 GM Nijmegen, The Netherlands; 3 Department of Pharmacy, Radboudumc, Nijmegen, The Netherlands; 4 Department of Rheumatology, Radboudumc, Nijmegen, The Netherlands

**Keywords:** Auto-immune rheumatic disease (AIRD), Biological DMARD, Dose loading, Pharmacodynamics / pharmacokinetics, Pharmaco-economics

## Abstract

**Background:**

Dose loading of biological disease modifying anti-rheumatic drugs (bDMARDs) in auto-immune rheumatic diseases (AIRDs) is performed to achieve steady state drug concentrations earlier after treatment start compared to dosing regimens without loading. Although loading inherently results in increased costs, treatment targets in terms of reduced disease activity may be achieved at an earlier state. It is an interesting topic that, surprisingly, has not received much attention in literature.

**Methods:**

In this review, we aimed at providing a theoretical description of the pharmacodynamic / -kinetic rationale for dose loading of bDMARDs in AIRDs and to systematically review the clinical evidence on the effectiveness of dose loading on disease activity in AIRDs.

**Results:**

Only a small number of studies (*n* = 5) has been published comparing the effectiveness of dose loading versus a regimen without dose loading of bDMARDs in AIRDs, addressing abatacept (*n* = 2), certolizumab pegol (*n* = 1), and secukinumab (n = 2). These studies provide insufficient evidence on superiority of dose loading in terms of disease activity compared to a dosing regimen without loading, while safety issues might be comparable.

**Conclusions:**

Although dose loading is commonly adopted for several bDMARDs in AIRDs, scientific evidence on its effectiveness and safety is surprisingly scarce and does not suggest superiority compared to a regimen without dose loading. More research in this field, also with regard to the pharmaco-economic consequences of dose loading, is urgently needed.

## Background

The introduction of biological disease-modifying anti-rheumatic drugs (bDMARDs) about two decades ago, resulted in significantly improved treatment outcomes and quality of life in patients with auto-immune rheumatic diseases (AIRDs), the most prevalent being rheumatoid arthritis (RA), psoriatic arthritis (PsA), and axial spondyloarthropathies (SpA). Tumor Necrosis Factor alpha inhibitors (TNFi’s) were the first to enter the market, and were followed by other targeted drugs such as IL-1 receptor antagonists, anti-IL-6R antibodies, IL-17 inhibitors, CTLA4 inhibitors, and anti CD20 antibodies (see Table 1). Efficacy and safety profiles have systematically been investigated. Safety data generally seem favourable and the drugs are well tolerated, although side effects such as a small increased risk of infection, tuberculosis reactivation and biological-specific adverse events are reported [1]. Another major ‘side effect’ of biologicals are their associated costs. For example, treatment with TNF alpha inhibitors in the Netherlands roughly ranges from €13.000 to €18.000 (www.medicijnkosten.nl), with even higher costs in the USA ($27.000 – $32.000) for a year of therapy [2].

**Table 1:** Overview of bDMARDs for AIRD, loading schedules (if applicable) and associated costs

Drug class	Drug	Terminal halve life	Authorised with dose-loading in AIRDs / other disease?	Loading schedule	Cost per patient year with loading dose^**a**^	Cost per patient year without loading dose^**a**^	Euro/ % increase medication costs due to dose-loading for full year use
TNFi	Adalimumab	14 days	No / Yes	NA			
	Certolizumab	14 days	Yes / NA	400 mg weeks 0,2,4, instead of 200 mg	€14,459	€12,965	€1495 / 11.5%
	Etanercept	3 days	No / Yes	NA			
	Golimumab	12 days	No / Yes	NA			
	Infliximab (RA)	9 days	Yes / Yes	3 mg/kg weeks 0,2,6 and 14, instead of 8 weekly^b^	€12,940	€11,323	€1617 / 12.4%
	Infliximab (PsA/axSpA)	9 days	Yes / Yes	5 mg/kg weeks 0,2,6 and 14, instead of 8 weekly^b^	€17,254	€15,097	€2157 / 12.5%
Anti-CD20	Rituximab	6–62 days	No / No	NA			
Anti CD80/86	Abatacept i.v.	14 days	Yes / No	750 mg weeks 0,2,4 instead of weeks 0,4^c^	€15,996	€14,853	€1143 / 7.1%
	Abatacept s.c.	14 days	Yes / NA	Start with 750 mg i.v. instead of once weekly sc^c^	€15,199	€14,056	€1143 / 7.5%
IL-1 receptor antagonist	Anakinra	5 h	No / No	NA			
Anti-IL6 receptor	Sarilumab	9 days (initial)	No / No	NA			
	Tocilizumab i.v.	3 days	No / No	NA			
	Tocilizumab s.c.	13 days	No / No	NA			
Anti-IL-17A	Secukinumab 150 mg	27 days	Yes / Yes	Weeks 0,1,2,3,4, instead of monthly	€9357	€7486	€1871 / 20%
	Secukinumab 300 mg	27 days	Yes / Yes	Weeks 0,1,2,3,4, instead of monthly	€18,714	€14,972	€3742 / 20%
	Ixekizumab	13 days	Yes / Yes	160 mg instead of 80 mg week 0	€16,116	€14,965	€1151 / 7.1%
Anti-IL-12/23	Ustekinumab	21 days	Yes / Yes	Additional injection at week 4^c^	€16,023	€12,819	€3204 / 20%

^a^www.medicijnkosten.nl, costs per patient year in the Netherlands

^b^ 3 mg/ and 5 mg/kg rounded to 3*100 mg and 4*100 mg vials per infusion

^c^ of the three weight based doses the middle dose of 750 mg was used

^c^ 45 mg (< 100 kg bodyweight) was used

Abbreviations: *i.v.* Intravenous, *s.c.* Subcutaneous, *TNFi* TNF inhibitor, *AIRDs* Auto-Immune Rheumatic Disease, *NA* Not Applicable, *AIRD* Auto-immune Rheumatic Diseases

Some biologicals, such as abatacept and infliximab, are administered using dose loading (i.e. higher dosing during treatment start) according to the Summary of Product Characteristics (SmPC), while others, such as etanercept and adalimumab, are applied without. The choice whether or not to advise a loading dose seems to be independent of the half-life of the bDMARD. Also, within a specific drug the use of dose loading often varies between indication, and dose loading is more often proposed, for example, for inflammatory bowel disease and psoriasis than for AIRDs (see Table 1). The use and rationale of dose loading of bDMARDs when starting treatment is therefore an interesting topic that, surprisingly, has not received much attention in literature, except for several pharmacokinetic modelling studies. The modelling studies provide us data on the potential effects of loading, but how this is translated to clinical outcome remains hypothetical.

The assumed rationale for dose loading is the achievement of steady state serum drug concentrations (Css) earlier after treatment start, hypothetically resulting in the achievement of treatment targets at an earlier stage. Dose loading is generally used when it is necessary to achieve effective concentrations as soon as possible, for example in the treatment of infections or cardiac arrhythmias. In AIRD, one could debate whether this is clinically relevant, especially since it may induce more (serious) side effects, and also induces higher medication costs.

In this narrative review, we will elucidate the rationale for dose loading of bDMARDs from a pharmacokinetic / -dynamic perspective, and we present a systematic review addressing the clinical evidence on the efficacy of dose loading on disease activity in patients with AIRDs.

## The rationale of dose loading of bDMARDs in AIRDs from a pharmacokinetic / -dynamic perspective

### The goal of dose loading

The main goal of dose loading is to reach an effective target steady state concentration (Css) at an earlier state, resulting in a faster clinical response. In pharmacokinetics, the Css refers to the situation where the overall intake of a drug is fairly in dynamic equilibrium with its elimination. In practice, it is generally considered that Css is reached after 4–5 times the half-life for a drug (T_1/2_). In some medical conditions, the time to attain Css after multiple doses of a drug is too long relative to the temporal demands of the condition being treated. Lidocain for example, which can be used to treat cardiac arrhythmias, has a T_1/2_ of 1–2 h. In this medical emergency, however, it is unacceptable to wait 4–10 h until Css is reached. In that case, it is therapeutically desirable to accelerate the time until the drug reaches the target concentration by giving a loading dose. By using a loading dose, the peak concentration is reached rapidly which is necessary to compete with clearance, so that the desired effect is achieved sooner [3]. Besides this pharmacokinetic rationale, other considerations for applying dose loading regimens are for instance when the medical condition results in high loss of the drug, such as in protein losing enteropathies in inflammatory bowel diseases, when the inflammatory load is high with subsequently high drug consumption in the first period, or when anti-drug antibodies have to be neutralised using more drug (i.e. non-linear kinetics). The latter phenomenon will lead to initial non-linear bDMARD clearance due to the presence of additional drug-binding proteins in the body, followed by linear pharmacokinetics when the surplus of these additional drug-binding proteins are all consumed. In fact, reversed Michaelis–Menten pharmacokinetics occur, as the original Michaelis–Menten pharmacokinetics is characterised by initial linear pharmacokinetics, followed by non-linear pharmacokinetics due to saturation of the enzyme system [4].

### How much loading dose is needed?

The amount of the loading dose is calculated by multiplying the desired peak concentration (Ctarget) by the volume of distribution of the drug (V_D_). In case of non-intravenous administration, the loading dose should also be corrected for the bioavailability (F) but it is mainly driven by the volume of distribution (V_D_) (loading dose = (C_target_ x V_d_) /F) [5]. This can cause practical problems with drugs with a high V_D_, as the calculated loading dose to achieve steady-state concentration may be impractically large. This is clearly illustrated with digoxin (T_1/2_: 30–40 h, V_D_: 83 l and F: 0,63, Ctarget: 0,8–2,0 μg/l): Based on the formula an initial oral dose of 740 μg is needed, but this has a relative high risk of side effects, and slow digitalization is warranted. Calculating the needed loading dose is even more complicated when loading is not applied for a pure pharmacological reason, but to compensate for loss of the drug or high drug consumptions in the early treatment phase, such as described above.

### The disadvantages of a loading dose

Despite the earlier achievement of Css, giving loading doses also has disadvantages. For example, sensitive individuals might abruptly be exposed to toxic concentrations and it can take a long time to reach lower concentrations when the drug has a long half-life. Furthermore, the need for both loading and maintenance doses creates complexity in prescribing, dispensing, administration, and monitoring of medication and this complexity increases the likelihood of human error. In the National Reporting and Learning System (NRLS), the use of loading doses indeed is associated with safety incidents [6, 7].

Exposure to more drug might also result in more bDMARD-related side effects such as infections. A review of Singh et al. [8] showed that infection risks are dose dependent, with higher risks in patients receiving supra-authorised dosing compared to standard dosing or sub- authorised dosing. The effects of loading doses were not included in the review of Sing and colleagues, unfortunately. However, as loading doses aim at reaching Css at an earlier stage, and not at increasing Cmax or Cavg (i.e. average plasma concentration at steady state) specifically, the increased infection risk may be minimal.

### Dose loading of bDMARDs

bDMARDs are large molecular weight (150 kDa) hydrophilic molecules which distribute predominantly in to lymphatic and blood vessels. Small amounts of bDMARDs also penetrate into the cells via fluid phase endocytosis or via receptor-mediated endocytosis via the (mostly neonatal) FcγR expressed on the membrane of immunological cells [9]. As a consequence, central volumes of distribution are small, approximately ranging from 2.4 to 6.5 l [4].

The half-lives of bDMARDs, however, vary widely from 70 h (etanercept) to 540 h (sarilumab). Inherently, the time to achieve an effective concentration and steady state also varies from days to weeks. From a purely pharmacokinetic perspective, dose loading can be used to shorten the time between the start of therapy and the timepoint when the minimal effective concentration is achieved, thus also achieving the pharmacodynamic effect and clinical response earlier. Given the relatively small volume of distribution, 1–2 additional regular doses as loading dose should be sufficient.

## Clinical evidence on the efficacy of dose loading of bDMARDs on disease activity in patients with AIRDs

### Methods

For studying clinical evidence on the efficacy of dose loading of bDMARDs on disease activity in patients with AIRDs, we systematically searched Pubmed / Medline, the Cochrane database, and clinicaltrials.gov to identify relevant studies until October 1st, 2018. Because of the anticipated lack of studies with high methodological quality, we aimed at including randomised controlled trials as well as other controlled studies with at least two treatment groups (i.e. with and without loading doses). A loading dose was defined as higher dosing during the first weeks of treatment compared to the period afterwards.

Eligible studies were performed with humans of at least 18 years of age, are written in English, with at least 20 patients with a follow-up of at least 3 months. The most commonly used biological agents, defined as receptor constructs or fusion proteins (suffix –cept), monoclonal antibodies (suffix –mab), and receptor antagonists (suffix -ra) approved for auto-immune rheumatic diseases (AIRDs; i.e. Rheumatoid Arthritis (RA), spondylarthropathies (Psoriatic Arthritis (PsA), spondyloarthropathies (SpA)) by the FDA and/or the EMEA, were included for review, which were: TNFi’s (infliximab, etanercept, adalimumab, certolizumab pegol, golimumab), anti CD20 (rituximab), anti-CD80/86 (abatacept), IL-1 receptor antagonist (anakinra), IL-6 inhibitors (sarilumab, tocilizumab), IL-17 inhibitors (secukinumab, ixekizumab), and anti-IL-12/23 (ustekinumab). Studies on dose loading of bDMARDs in other conditions than AIRD were excluded (see Appendix A).

Identified references were independently screened for eligibility by title and abstract by two reviewers (GG, LV). Full text papers were then used to decide on eligibility. In case of discrepancies, a third reviewer (AB) was consulted. The reference lists of included articles were then studied to further identify relevant papers (snowballing). Conference proceedings and reports of meetings were also considered for inclusion. Search strings and strategies are provided in Appendix A.

Data on patient population, sample size, study design, dosing regimens, main outcome measures, and secondary outcome measures / safety issues were extracted from the selected papers. We did not plan a formal quantitative meta-analysis because of the anticipated clinical heterogeneity and low numbers of included studies per drug.

### Results

**Fig. 1:**
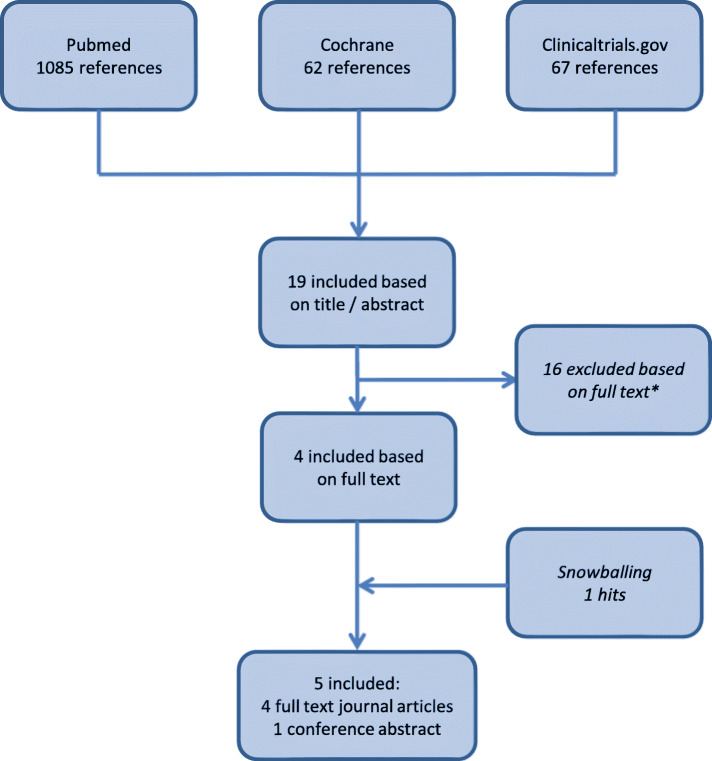
Search results and inclusion. * Reasons for exclusion: no comparison loading dose versus no loading dose (*n* = 15), (pharmacodynamic/−kinetic) modelling studies instead of real data (*n* = 2) and a narrative review (n = 1)

Results from the search procedures are listed in Fig. 1.

Five studies were included in this review; four full text journal articles and one conference abstract. The selected studies were published between 2011 and 2018. Two [10, 11] comprised post hoc analyses of previously performed large trials. The other three studies [12–14] were randomised controlled trials. Two were performed on PsA [12, 13], two on RA [10, 11], and one on SpA (Ankylosing Spondylitis, AS) [14]. Two studies described the effect of dose loading of abatacept [11, 12], one of certolizumab pegol [10], and two of secukinumab [13, 14]. Table 2 lists the main findings for each of these studies.

**Table 2:** Included references and results

Reference	Patient population	Study design	Drug	Groups and dosing regimens	Numbers of patients	Primary outcome	Results primary outcome	Relevant secondary outcomes	Results secondary outcomes
Mease et al., 2011 [12]	Psoriatic Arthritis	Randomized, double-blind, placebo-controlled Phase II trial	Abatacept	Group 1: 3 mg/kg at days 1, 15, and 29, every 4 weeks thereafter Group 2: 10 mg/kg at days 1, 15, and 29, every 4 weeks thereafter Group 3: 30 mg/kg at days 1 and 15, 10 mg/kg at day 29 and every 4 weeks thereafter^a^	Group 1: 43 Group 2: 40 Group 3: 45	ACR20 at 6 months	ACR20 Group 1: 33% Group 2: 48% Group 3: 42% No significant differences reported between the groups (especially groups 2 and 3)	HAQ-DI SF-36 ACR50 ACR70 Damage of joints on MRI AE’s	No significant differences reported between the groups (especially groups 2 and 3)
Schiff et al., ACR meeting 2012 [11]	Rheumatoid Arthritis	Randomized study; post-hoc analysis on data from the ACQUIRE and AMPLE studies	Abatacept	Group 1: s.c. 125 mg/week (ACQUIRE) Group 2: s.c. 125 mg/week, plus i.v. loading dose 10 mg/kg on day 1 (AMPLE)	Group 1: 736 (ACQUIRE) Group 2: 318 (AMPLE)	ACR20 at weeks 2, 4, 8, 12, 16, 20, 24.	ACR20 at weeks 2, 4, 8, 12, 16, 20, 24 Group 1: 27.4, 42.5, 58.5, 60.1, 66.0, 70.1, 66.0% Group 2:24.6, 44.5, 58.0, 66.6, 69.3, 72.4, 74.8% No significant difference between the groups	HAQ-DI at weeks 2, 4, 8, 12, 16, 20, 24 Changes in DAS28 CRP from baseline over 6 months	No significant difference between the groups
Takeuchi et al., Mod Rheumatol 2016 [10]	Rheumatoid Arthritis	Open label extension study; post hoc analysis of the J-RAPID and HIKARI trials	Certolizumab pegol (CZP)	Group 1: 400 mg loading dose at weeks 0, 2, and 4, then 200 mg Q2W thereafter Group 2: 200 mg Q2W	Group 1: 198 Group 2: 160	ACR20 at weeks 4, 8, 12, 16, 20, 24% Low disease activity	ACR20 at week 4 Group 1: 62.2 and 67.2% Group 2: 57.1 and 49.5% ACR20 at week 8 Group 1: 82.9 and 71.6% Group 2: 69.6 and 61.1% Absolute values for week 12, 16, 20, and 24 were not provided; graphical presentation only. No statistical data provided^b^	Not well defined in methods section, but reported for: ACR50,70 responses % Low disease activity (LDA) at weeks 12 and 24 Plasma concentrations of CZP and antibodies against CZP Adverse Events rates	Loading dose groups showed faster ACR responses followed by sustained ACR responses up to 24 weeks compared to patients who did not receive loading dose. Higher levels of antibodies in group without loading dose. Similar adverse event rates. No statistical data provided♠
Mease et al., Ann Rheum Dis, 2018 [13]	Psoriatic Arthritis	Randomised double-blind phase III FUTURE 5 study	Secukinumab	Group 1: 150 mg s.c. with loading dose, at weeks 0, 1, 2, 3 and 4, then every 4 weeks thereafter Group 2: 150 mg s.c. without loading dose, at weeks 0, (at 1, 2, 3 placebo) and 4, then every 4 weeks thereafter^a^	Group 1: 222 Group 2: 220	ACR20 at week 16	ACR20 response at week 16 Group 1: 55.5% Group 2: 59.5% No statistical difference between loading versus no loading	Radiographic progression at week 24 (van der Heijde-modified total Sharp score) HAQ-DI DAS28-CRP ACR50/70 response Proportion of patients achieving MDA at week 16 AE’s and SAE’s	No statistical difference between loading versus no loading
Kivitz et al., Rheumatol Ther, 2018 [14]	Ankylosing Spondylitis	Randomized placebo controlled trial MEASURE 4 study	Secukinumab	Group 1: 150 mg at week 0 and every 4 weeks thereafter, with loading dose 150 mg at weeks 1, 2, and 3 Group 2: 150 mg at week 0 and every 4 weeks thereafter, with placebo at weeks 1, 2, and 3	Group 1: 116 Group 2: 117	ASAS20 at 16 weeks	ASAS20 at 16 weeks Group 1: 59.5% Group 2: 61.5% No significant difference.	ASAS20 at 52, and 104 weeks ASAS40 at 16 weeks % achieving ASAS20 and ASAS40 Change from baseline in BASDAI % AEs, % SAEs	No significant difference

^a^ Placebo groups were removed from the table [12, 13]

^b^ Exact patient numbers in each group at each time point could not be extracted from the manuscript for post hoc statistical analysis

Abbreviations:

*ACR* American College of Rheumatology (ACR20 means 20% improvementin tender or swollen joint counts as well as 20% improvement in three of the other five criteria; ACR50 70 analogous)

*HAQ-DI* Health Assessment Questionnaire, Disability Index

*SF36* Short Form 36

*ASAS* Assessment in Spondyloarthritis International Society

*Q2W* Every two weeks

*DAS28 CRP* Disease Activity Score based on 28 joints and C-reactive protein

*BASDAI* Bath Ankylosing Spondylitis Disease Activity Index

*MDA* Minimal Disease Activity

*(S)AE* (Serious) Adverse Events

*CZP* Certolizumab pegol

### Primary outcome: ACR20 / ASAS20

Primary outcome measure was the ACR20 response in four of the five studies. and ASAS20 in the remaining study. For abatacept, different loading dose regimens were applied. In the study of Mease et al. [12], loading was performed by dosing (3 mg/kg, 10 mg/kg, or 30 mg/kg) every 2 weeks for the first 3 times, followed by 3, 10 and 10 mg/kg four-weekly respectively. Schiff et al. [11] added a single dose of 10 mg/kg intravenously at day one, followed by a 125 mg/week subcutaneous dose. For patients with PsA loading was reported not to enhance efficacy in terms of ACR20 response compared to without loading dose [12]. Comparably, in RA, loading appeared not to affect time to onset and magnitude of ACR20 response [11].

For certolizumab pegol only one study was included [11], evaluating the ACR20 response at week 16 in patients with RA with and without loading dose of 400 mg at weeks 0, 2, and 4, with regular doses of 200 mg every other week afterwards. The ACR20 response in patients with a loading dose seemed (numerically) to be achieved faster and sustained until 24 weeks follow-up compared to those receiving no loading dose (no data on statistical significance reported).

In two studies, secukinumab was dosed 150 mg sc every 4 weeks starting at week 0, with loading doses at weeks 1, 2, and 3 compared to patients without loading dose. In patients with PsA [13] or with AS [14], loading did not contribute to improved outcome in terms of ACR20 response at 16 weeks compared to no loading.

### Secondary outcomes and safety issues

Secondary outcomes were abundantly reported in these studies. For the studies reporting on abatacept, improvements were described on secondary outcome measures compared to placebo, but no statistical difference was observed between the groups receiving a loading dose compared to the group not receiving a loading dose. For certolizumab pegol, lower antibodies levels were found in those receiving a loading dose. No differences were found in the secondary outcome measures reported between those who did and did not receive loading doses of secukinumab.

(Serious) Adverse Event rates were low. None of the five studies reported statistically significant differences when comparing loading versus no loading.

## Discussion and conclusions

This paper highlights two important aspects of dose loading of bDMARDs in AIRDs. First, dose loading seems to be a sensible approach from a pharmacokinetic perspective. However, hardly any clinical evidence has been published on this topic, and studies that did address this topic were heterogeneous in terms of patient population and drug under investigation, and generally from moderate methodological quality. Safety profiles seem favorable, but, again, data is scarce. As a consequence, evidence on superiority in terms of disease activity compared to a dosing regimen without loading is lacking, although safety profiles may .

The small number of studies identified comparing regimens with dose loading to regimens without, does not seem to be caused by a suboptimal search, as broad criteria were used and a systematic search was done in the main scientific medical databases without limits on the date of publication. This review being narrative systematic or scoping in nature, we decided to systematically search a limited number of databases. From this perspective, we chose not to include the EMBASE database. It is unlikely that searching EMBASE would have changed the conclusions of this paper, especially since the level of agreement with Pubmed/Medline, the Cochrane database, and clinicaltrial.gov is considerable, and we used the snowballing technique to identify papers that might have been missed in the performed searches or might only have been found in other databases. We could have expanded the number of selected studies for this review if we had included pharmacokinetic / -dynamic (PK / PD) modelling studies on dose loading. For example, Ternant and colleagues [15] suggested after PK / PD modelling that dose loading of adalimumab may lead to increased benefit for patients with RA. These modelling studies provide indirect evidence for clinical efficacy and provide inspiring new insights and ideas for clinical research, but they do not, however, prove clinical efficacy. Other sources of indirect evidence favoring dose loading are drug plasma concentrations and antibody studies, sometimes with short term efficacy. Yamamoto et al. [16] reported lower antibodies to certolizumab pegol in patients receiving a loading dose compared to those who did not. However, no statistical evidence for this statement was provided. In patients with Crohn’s disease treated with a loading dose of adalimumab, Karmiris et al. [17] reported higher trough concentrations after 4 weeks after starting treatment, less frequent non-response, and longer sustained benefit than the patients who did not receive a loading dose. However, although lower antibody levels and higher plasma concentrations and better 4 week clinical efficacy are promising results, in our view clinical efficacy at weeks 12 and 24 is a more relevant clinical outcome. Also, this study included patients not with AIRD, but with inflammatory bowel diseases (IBD). Summarizing, PK / PD (modelling) studies report indirect evidence and whether these findings can be translated to better clinical outcome remains unclear. For this reason, these studies were not included in the current review.

Our work focused on AIRDs alone and the question is whether the effects of dose loading are comparable to other disease entities in which bDMARDs are prescribed, such as in psoriasis, IBD, or ophthalmological conditions. In psoriasis, for example, a review has been performed describing the efficacy and safety of dose loading of etanercept and adalimumab [18]. This study concluded that for etanercept dose loading resulted in more rapid and higher percentages of patients showing skin improvements, while for adalimumab there was insufficient evidence. In Crohn’s disease, the additional benefit of a loading dose of 160 mg adalimumab has been demonstrated in several studies [17–19], and this regimen is also recommended by the FDA. Dose loading may thus be more appropriate in non-AIRDs than in rheumatic inflammatory diseases. This may be explained by essential pathophysiological and background treatment differences. For example, in IBD the bioavailability of the drug might sometimes be lower due to protein losing enteropathy. The total inflammatory load might also be different between AIRDs and other conditions, especially when comparing RA with IBD, necessitating more drug in the active initial phase. Finally, in RA, a bridging treatment is performed using glucocorticoids orally or intramuscularly, This bridging reduces symptoms of AIRDs rapidly, and is especially relevant in the light of the ‘window of opportunity’. This window represents a timeframe in which the disease is potentially amenable and may be reset; aggressive therapy administered in this window can slow the rate of structural damage at long term [20]. Bridging with glucocorticoids negates a possible effect of dose loading, and is proven efficacious and less expensive. However, initial glucocorticoids treatment has not formally been used in the clinical studies we identified, and also, in IBD this is applied at least as frequent as in RA and more than in SpA. When summarizing the considerations above, the rationale for dose loading in AIRDs seems absent, but for IBD or psoriasis it might be a beneficial strategy.

Dose loading aims at achieving Css at an earlier state, and not increasing the maximal or average plasma concentration at steady state. This implies that safety issues were assumed not to be a major concern in dose loading. Indeed, we did not identify significantly increased adverse events in this review. Although large scale data between treatment strategies with or without dose loading are absent, similar results were reported for dose loading of adalimumab in Crohn’s disease [19], further supporting our assumption of its safety.

Besides considering clinical evidence and safety, another important issue is the economic aspect of dose loading. To our knowledge, no cost effectiveness or budget impact analyses comparing biological treatment with and without dose loading have been performed. Hypothetically, there are two effects in which dose loading might contribute to higher cost and (with equal effectiveness) lower cost effectiveness. Firstly, the loading dose itself leads to increased medication use and cost. The magnitude of this is an increase between 7.1 and 20% for loading compared to no loading, for the first full year of treatment compared to subsequent years (as can be derived from Table 1). Although this increase in cost regresses when the patient stays on the drug longer than the first year, this is a limited effect while there is a substantial amount of patient who stop the drug after for example 3 to 6 months of use due to lack of efficacy or adverse effects. Recent developments in state of art bDMARD care, such as treat to target, will further shorten drug survival and herewith further increase the relative cost impact of dose loading. Secondly, dose loading might also lead to more ‘dose creep’. This is the effect that health care providers increase the dose in non-responding patients above the authorised and maximum effective dose [21]. Although this is not effective and indeed not officially endorsed, the phenomenon has been well recognised. Further research on this topic is warranted including a comprehensive cost-effectiveness analysis. These results are needed for evidence based recommendations with regard to dose loading.

In conclusion, although dose loading seems reasonable from a pharmacodynamic / - kinetic perspective, there is insufficient evidence on its superiority in terms of disease activity compared to a dosing regimen without loading. Data on cost-effectiveness of these regimens have not been published, while safety issues might be comparable. More research on this topic is urgently needed, based on which authorities may reconsider reimbursement of dose loading regimens to further encourage evidence based practice.

## Supplementary information

**Additional file 1.**

## Data Availability

Not applicable.
