# Supplementary material for: The pharmacological and clinical aspects behind dose loading of biological disease modifying anti-rheumatic drugs (bDMARDs) in auto-immune rheumatic diseases (AIRDs): rationale and systematic narrative review of clinical evidence
Source: BMC Rheumatol. 2020 Jul 28;4:37. doi: 10.1186/s41927-020-00130-x (PMC7385956; doi:10.1186/s41927-020-00130-x)
Supplement: Supplementary file 1 — Additional file 1. [file 41927_2020_130_MOESM1_ESM.zip › Geurts et al._Appendix A.docx]

**Appendix A**

**Search Pubmed**

Search performed at 15-9-2018 (1085 references)

(((arthritis, rheumatoid[MeSH Terms]) OR arthritis, rheumatoid[Title/Abstract]) OR ((Spondylarthropathies[MeSH Terms]) OR Spondylarthropathies[Title/Abstract]) OR ((Spondylitis, Ankylosing[MeSH Terms]) OR Spondylitis, Ankylosing[Title/Abstract]) OR ((Arthritis, Psoriatic[MeSH Terms]) OR Arthritis, Psoriatic[Title/Abstract])) AND ((((((Tumor Necrosis Factor-alpha/administration and dosage[MeSH Terms])) OR Tumor Necrosis Factor-alpha/drug effects[MeSH Terms]) OR Tumor Necrosis Factor-alpha/therapeutic use[MeSH Terms]) OR Tumor Necrosis Factor-alpha[Title/Abstract]) OR (((biological[Title/Abstract]) OR biologic[Title/Abstract]) OR bDMARD[Title/Abstract]) OR ((Certolizumab Pegol[MeSH Terms]) OR Certolizumab Pegol[Title/Abstract]) OR ((Etanercept[MeSH Terms]) OR Etanercept[Title/Abstract]) OR ((Adalimumab[MeSH Terms]) OR Adalimumab[Title/Abstract]) OR ((golimumab[MeSH Terms]) OR golimumab[Title/Abstract]) OR ((IL-6 inhibitor[MeSH Terms]) OR IL-6 inhibitor[Title/Abstract]) OR ((tocilizumab[MeSH Terms]) OR tocilizumab[Title/Abstract]) OR ((sarilumab[MeSH Terms]) OR sarilumab[Title/Abstract]) OR ((IL-17a antagonist[MeSH Terms]) OR IL-17a antagonist[Title/Abstract]) OR ((secukinumab[MeSH Terms]) OR secukinumab[Title/Abstract]) OR ((ixekizumab[MeSH Terms]) OR ixekizumab[Title/Abstract]) OR ((Interleukin 1 Receptor Antagonist Protein[MeSH Terms]) OR Interleukin 1 Receptor Antagonist Protein[Title/Abstract]) OR ((Anakinra[MeSH Terms]) OR Anakinra[Title/Abstract]) OR ((CD20 antibody[MeSH Terms]) OR CD20 antibody[Title/Abstract]) OR ((Rituximab[MeSH Terms]) OR Rituximab[Title/Abstract]) OR ((IL-12/23 inhibitor[MeSH Terms]) OR IL-12/23 inhibitor[Title/Abstract]) OR ((Ustekinumab[MeSH Terms]) OR Ustekinumab[Title/Abstract]) OR ((CTLA4 protein[MeSH Terms]) OR CTLA4 protein[Title/Abstract]) OR ((Abatacept[MeSH Terms]) OR Abatacept[Title/Abstract]) OR ((infliximab[MeSH Terms]) OR infliximab[Title/Abstract])) AND (((("Biological Therapy/administration and dosage"[MeSH Terms])) OR Biological Therapy/pharmacology[MeSH Terms]) OR ((dosing regimen[MeSH Terms]) OR dosing regimen[Title/Abstract]) OR ((loading dose[MeSH Terms]) OR loading dose[Title/Abstract]) OR ((Dose-Response Relationship, Drug[MeSH Terms]) OR Dose-Response Relationship[Title/Abstract]) OR ((Drug Administration Schedule[MeSH Terms]) OR Drug Administration Schedule[Title/Abstract]))

**Search “Clinicaltrials.gov”**

Searched for ‘condition’ and ‘other term’ at 15-9-2018

(Arthritis OR Spondyl(o)arthropathy OR Spondyl(o)arthropathies OR Ankylosing spondylitis)

AND (Loading dose OR Dosage OR Dosing regimen OR Drug administration schedule)

12 separate searches

77 hits, 10 duplicates 🡪 67 unique references

**Search Cochrane library**

Search performed at 15-9-2018

("rheumatoid arthritis":ti,ab OR "ankylosing spondylitis":ti,ab OR "spondylarthropathies":ti,ab OR "spondyloarthropathies":ti,ab OR "psoriatic arthritis":ti,ab) AND ("dose loading":ti,ab OR "dosing regimen":ti,ab OR "dose response relationship":ti,ab OR "drug administration schedule":ti,ab)

62 unique references
